# Cross‐sectional survey shows that follow‐up formula and growing‐up milks are labelled similarly to infant formula in four low and middle income countries

**DOI:** 10.1111/mcn.12269

**Published:** 2016-04-15

**Authors:** Catherine Pereira, Rosalyn Ford, Alison B. Feeley, Lara Sweet, Jane Badham, Elizabeth Zehner

**Affiliations:** ^1^ JB Consultancy Bryanston South Africa; ^2^ Helen Keller International Washington D.C. USA

**Keywords:** Breastmilk substitutes, infant formula, follow‐up formula, growing‐up milk, labelling, infant and young child feeding

## Abstract

This cross‐sectional survey assessed the characteristics of labels of follow‐up formula (FUF) and growing‐up milk (GUM) compared with infant formula (IF), including cross‐promotion practices between FUF/GUM and IF manufactured by the same company, sold in Phnom Penh, Cambodia; Kathmandu Valley, Nepal; Dakar Department, Senegal; and Dar es Salaam, Tanzania. All products were imported. A wide recommended age/age range for introduction was provided by manufacturers across all sites, with products with an age recommendation of 0–6 months being most prevalent in three sites, representing over a third of all products. Various age categories (e.g. 1, 1+ and Stage 1) commonly appeared on labels. A number of descriptive names (e.g. infant formula and milk formula) per category of age of introduction were used with some appearing across more than one category. Images of feeding bottles were found on most labels across all age categories, but prevalence decreased with older age categories. The majority of FUF/GUM manufactured by IF companies across all sites displayed at least one example of cross‐promotion with one or more of the company's IF: two‐thirds or more contained similar colour schemes/designs and similar brand names; 20–85% had similar slogans/mascots/symbols. A wide and potentially confusing range of ages/categories of introduction and descriptive names were found, and cross‐promotion with IF was common on FUF/GUM labels. Global guidance from normative bodies forms the basis of most low and middle income countries policies and should provide specific guidance to prohibit cross‐promotion between FUF/GUM and IF, and all three categories should be classified as breastmilk substitutes.

**Key messages**

- A range of ages of introduction and descriptive names are commonly found on labels of commercial milks for infants and young children in four low and middle income countries.
- Cross‐promotion between a manufacturer's infant formula and follow‐up formula and/or growing‐up milk is common practice in four low and middle income countries.
- Normative bodies should provide detailed guidance to prohibit practices whereby follow‐up formula and growing‐up milks, marketed for the age range 6–24 months, can be compared, even indirectly, with infant formula.
- All commercial milks for infants and young children should be classified as breast milk substitutes and therefore be required to comply with the International Code of Marketing of Breast‐milk Substitutes.

## Introduction

Globally, much attention is being paid to the impact that feeding practices have on the nutritional status, growth and development, health and thus the very survival of infants and young children (IYC) (WHO 2003). As a consequence, infant and young child feeding (IYCF) practices and the promotion of foods marketed as suitable during the critical first 2 years of life are under scrutiny (Codex Alimentarius 2012; WHA 2012).

The market for commercial milk marketed for IYC (CM), which includes infant formula (IF), follow‐up formula (FUF) and growing‐up/toddler milk (GUM; see Box 1), was worth US$41 billion in 2013 (Kent 2015). Strong market growth is being experienced in low and middle income countries (LMIC), with the biggest growth in China (+12.3 billion USD) followed by Eastern Europe (+2.0 billion USD), Latin America (+1.7 billion USD) and the Middle East and Africa (+1.5 billion USD) (Coriolis 2014). One‐third of the global spend on CM in 2013 was attributed to GUM, making it the largest CM category (Bandy 2014). A report by the European Food Safety Authority (EFSA) in 2013 found 244 GUM available on the market in European Union Member States. In 10 out of the 12 countries, the retail market for GUM increased in volume between 0.6% (Switzerland) and 11.6% (the Netherlands) over the previous year, and the weighted average of the retail market value was €78.76 m (EFSA 2013). The growth of the CM market is considered to be linked to increasing disposable incomes and the increasing numbers of working women (UBIC Consulting 2014). This growth is concerning considering the position taken by the World Health Assembly that FUF is not necessary (WHO 1986) and is unsuitable when used as a replacement for breastmilk from 6 months onwards (WHO 2013), together with the drive to increase exclusive (to 6 months) and continued (to 2 years and beyond) breastfeeding (WHA 2012).

Evidence shows that FUF/GUM advertisements are perceived by mothers as promoting IF (Berry *et al*. 2010; Smith & Blake 2013; Cattaneo *et al*. 2014) and that IF, FUF and GUM are seen collectively as ‘formula’ (Berry *et al*. 2012a). In the United Kingdom, for example, 40% of women surveyed (*n* = 2000) believed that there were no differences between the different CM categories (NOP World 2005). This perception is largely attributed to the marketing practice of ‘line extension’ and a focus on ‘brand advertising’, resulting in IF, FUF and GUM appearing similar or the same to consumers. This deliberate strategy enables manufacturers to evade national restrictions on the advertising of IF and in some cases FUF/GUM (Berry *et al*. 2012a; Smith & Blake 2013; Cattaneo *et al*. 2014). The Codex Alimentarius Committee on Nutrition and Foods for Special Dietary Uses (CCNFSDU) is currently undertaking a full review of the current Follow‐Up Formula Standard (Codex Alimentarius 1987). At the 2014 meeting of the CCNFSDU, the WHO raised a concern about the continuing marketing practices for FUF, which they stated were undermining both exclusive and continued breastfeeding in many countries and further requested that the review ‘include clear language as to the need for strong regulatory measures to avoid inappropriate marketing of FUF, not only through necessary labelling requirements, but in line with the marketing restrictions on breastmilk substitutes, as reflected in the International Code’ (Codex Alimentarius 2014). The adverse effects of marketing of IF on exclusive breastfeeding rates (Foss & Southwell 2006) and duration of breastfeeding have been documented (Foss & Southwell 2006; Sobel *et al*. 2011; Smith & Blake 2013).

Previous studies undertaken to assess the impact of CM marketing have included questionnaires and qualitative interviews with mothers and assessments of print adverts (Berry *et al*. 2010; Cattaneo *et al*. 2014). Although these studies have highlighted similarities in the packaging, branding and labelling as a contributing factor to consumers not being able to distinguish between different categories of CM, none have systematically/quantitatively assessed the labelling practices of FUF and GUM against IF. The aim of this research was to examine the characteristics – including age of introduction, descriptive names, use of the term ‘formula’ and images of bottles/teats – on the labels of FUF and GUM available for sale in the most populous city/metropolis in four countries (Cambodia, Nepal, Senegal and Tanzania), in order to determine same/similar characteristics and cross‐promotion with IF manufactured by the same company.

Box 1 **Definitions**

**Commercial milk for infants and young children** (**CM**): Means any commercially produced milk indicating on its label that the product is intended for children younger than 2 years of age (the minimum recommended breastfeeding period), even if the upper age recommendation on the product label indicates its suitability for children older than 2 years of age, including the following:

- Infant formula (IF);
- Follow‐up formula (FUF);
- IF/FUF for special dietary or medical purposes;
- Growing‐up/toddler milks (GUM);
- Any other milk or milk‐like drinks (in liquid or powdered form to be reconstituted) marketed or otherwise represented as suitable for feeding children younger than 2 years of age by:
  - Using the words baby/babe/infant/toddler/young child in the context of a child's age,
  - Recommending an age of introduction less than 2 years on the label, or
  - Using an image of a child appearing younger than 2 years of age or an image/text of infant feeding (which could include a bottle).

Commercial milk for infants and young children therefore includes, but is not limited to, breast milk substitutes as defined by the International Code of Marketing of Breastmilk Substitutes.^*^ It however excludes the following: meal replacements; nutrient supplements (e.g. drops); breast milk fortifier; milk or milk‐like products for general consumption/older children that are not marketed as suitable for feeding children younger than 2 years; and products not available to customers through retail/wholesale outlets (e.g. products only distributed through government/humanitarian programmes or products only available for purchase online).

**Infant formula** (**IF**): Means a breast milk substitute specially manufactured to satisfy, by itself, the nutritional requirements of infants during the first months of life up to the introduction of appropriate complementary feeding (Codex Alimentarius 1981).

**Follow‐up formula** (**FUF**): Means a food intended for use as a liquid part of the weaning diet for the infant from the sixth month on and for young children (12–36 months). It is a food prepared from the milk of cows or other animals and/or other constituents of animal and/or plant origin, which have been proved to be suitable for infants from the sixth month on and for young children (Codex Alimentarius 1987).

**Growing‐up**/**Toddler milk** (**GUM**): Growing‐up milks, ‘toddlers' milks’ or similar products intended for children aged 1–3 years, usually to replace cows' milk, include but are not limited to drinks (either in liquid form or powder form to be reconstituted) based on cow, goat or sheep milk or originating from soy, rice, oats or almonds with or without modification of the protein composition or content and supplementation of fatty acids, micronutrients or other substances with a potential nutritional effect, such as probiotics, prebiotics or symbiotics (EFSA 2013).

^*^Any food being marketed or otherwise represented as a partial or total replacement for breast milk, whether or not suitable for that purpose (WHO, 1981).

## Methods

### Study design and research setting

In this cross‐sectional survey, CM available for sale in the most populous city in Cambodia and Tanzania and in the largest metropolitan areas in Nepal and Senegal were purchased, and the information on their labels were captured and assessed. This formed part of a larger study that set out to collect data on a range of IYCF products, the results of which are reported elsewhere (Sweet *et al*. 2016). Data collection was conducted in Phnom Penh, Kathmandu Valley, Dakar Department and Dar es Salaam, representing 10% (National Institute of Statistics Cambodia 2011), 6% (Ministry of Health and Population Nepal 2012), 21% (Agence Nationale de la Statistique et de la Démographie Senegal 2010) and 7% (National Bureau of Statistics Tanzania 2011) of the countries' populations, respectively.

**Figure 1:**
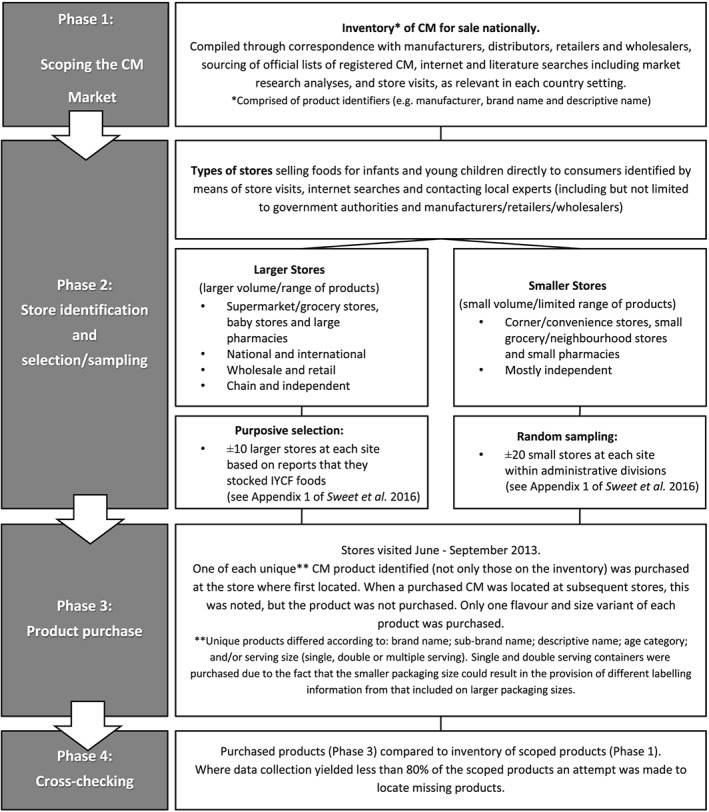
Data collection process.

Figure 1 presents a flow diagram of the data collection process. Phase 1, a scoping phase to create an inventory of CM available nationally, was designed to determine whether the product purchase conducted in each site (Phase 3) yielded at least 80% of the products theoretically available. A cross‐checking (Phase 4) was used to determine whether the 80% had been reached or whether additional product purchase was necessary. Phase 2 involved store identification and selection/sampling. It was anticipated that the majority of products manufactured by large/medium enterprises and sold nationally could be purchased from a purposive selection of larger store types (Sweet *et al*., 2012), while products manufactured by local small and medium enterprises, which might not be distributed through formal distribution channels, could be purchased from smaller stores. Smaller stores, of which there were many, were randomly sampled as it was not possible to obtain a list of all smaller stores per study site.

### Data extraction, entry and analysis

The Codex Alimentarius definition of a product label as ‘any tag, brand, mark, pictorial or other descriptive matter, written, printed, stencilled, marked, embossed or impressed on, or attached to, a container of food’, was used in this study (Codex Alimentarius 1985) and excludes packaging inserts defined as ‘printed information (excluding the product label) that is inserted into the product or affixed to it’ (WHO 1981). Product labels were photographed or scanned and uploaded to a central digital folder. Label text in English, French or the official national language (Khmer in Phnom Penh, Nepali in Kathmandu Valley, French in Dakar Department or Swahili in Dar es Salaam) was assessed. In Kathmandu Valley and Dar es Salaam, all labels had text in English, and in Dakar Department and Phnom Penh, respectively; professional translation of 36 French labels and 30 Khmer labels to English was required. All text of labels in Kathmandu Valley, Dakar Department and Dar es Salaam was assessed. In Phnom Penh 75% of labels had all text assessed, while the remaining 25% (*n* = 28) of labels had some text assessed. Twelve labels in Phnom Penh had text in a language that was not assessed. Recommended age/age range of introduction was determined using other methods such as Google translate in order to categorise products to ensure full sample representation.

One researcher carried out data extraction by entering all predetermined categories of descriptive data from the product label into Microsoft Excel 2010 (Microsoft Corporation, Redmond, WA, USA). The allocation of manufacturer's name can be complex, because in some countries certain manufacturers have acquired other manufacturers or a different manufacturer's brand/s. For example, Wyeth was acquired by Pfizer in 2009 1Thomson Financial (2009). PFE – Pfizer to Acquire Wyeth, Creating the World's Premier Biopharmaceutical Company. [online] Available at: http://www.pfizer.com/files/investors/presentations/q4_transcript_012609.pdf [Accessed 16 Jun. 2015]. and in 2012 Nestlé purchased Pfizer's infant nutrition division including Wyeth and associated brands. 2Nestlé (2009). Nestlé to acquire Pfizer Nutrition in strategic move to enhance its position in global infant nutrition. [online] Available at: http://www.nestle.com/media/pressreleases/allpressreleases/april-2012-nutrition-announcement [Accessed 16 Jun. 2015]. Therefore, in this study, the manufacturer and brand names captured and reported were those provided on the product label.

For eight fields (related to descriptive name and cross‐promotion practices), two researchers independently completed the data entry/extraction for the data extraction key for CM labels. Disagreements regarding information extracted were resolved by consensus, and where consensus could not be reached (4.8% of answers for Phnom Penh; 5.8% of answers for Kathmandu Valley, 2.8% of answers for Dakar Department and 6.3% of answers for Dar es Salaam), a third researcher made the final decision in consultation with the first and second researchers. Label information from the data extraction database was used to complete the CM labelling practices checklist, which included questions related to recommended age/age range for use and cross‐promotional practices. The use of the term ‘formula’ and images of feeding bottles/teats was also assessed. For the purpose of this study, key label elements that were assessed are defined in Box 2.

Simple frequencies were calculated in Excel, and descriptive statistics were used to present a record of current labelling practices.

Box 2 **Definition of label elements assessed in this study**

**Age of introduction**: The lowest age, in months or years, recommended on the label of commercial milks as being the appropriate age from which the product can be consumed.

**Age category**: Descriptors in words (e.g. for baby/toddler) and/or numbered stages (e.g. Stage 1, 2 and 3), but not the representation in months and years, that appears on the label of commercial milks to indicate the appropriate life stage for the product's use.

**Descriptive name**: A name on the front of the label providing a description of the commercial milk, which is sufficiently clear to enable consumers to know its true nature and distinguish it from other products with which it might be confused (e.g. IF or complementary food).

**Cross‐promotion**: A form of marketing promotion where customers of one product are targeted with promotion of a related product. In this study, it was assessed as being when a manufacturer of a range of commercial milks made a link between products with an age of introduction of 6–24 months with a product/s with an age of introduction of 0–6 months, based on the presence of a similarity between two products' colour schemes or designs; names; and/or slogans, mascots, or symbols. Cross‐promotion was also deemed to have occurred, if a product for the age group 6–24 months referred to the same manufacturer's IF by displaying an image of a pack‐shot (with or without accompanying text) or including a textual reference that recommends by name the use of the IF. The presence of the manufacturer's name or corporate logo alone was not deemed as cross‐promotion.

## Results

### Descriptive characteristics and manufacturer information

The number of CM products purchased in each of the four sites was 112 in Phnom Penh (IF, *n* = 43; FUF, *n* = 32; GUM, *n* = 25; unclear, *n* = 12), 14 in Kathmandu Valley (IF, *n* = 5; FUF, *n* = 5; GUM, *n* = 4), 36 in Dakar Department (IF, *n* = 16; FUF, *n* = 15; GUM, *n* = 5) and 22 in Dar es Salaam (IF, *n* = 12; FUF, *n* = 5; GUM, *n* = 5).

The greatest number of manufacturers were found in Phnom Penh (*n* = 25). In Kathmandu Valley, three manufacturers were represented; in Dakar Department nine; and in Dar es Salaam, eight. Nestlé products were found in all four sites, and Danone products in three (excluding Phnom Penh). The three manufacturers with the greatest number of products available in the four study sites, as reflected by the company name on the label, were as follows: Friesland Campina (10.7%), Mead Johnson and Company (8.0%) and Guigoz Laboratories (7.1%) in Phnom Penh; Nestlé (71.4%), Danone (14.3%) and SMA Nutrition (14.3%) in Kathmandu Valley; Danone (30.6%), Nestlé (22.2%) and Regilait (16.7%) in Dakar Department; and Nestlé (18.2%), Wyeth Nutritionals (18.2%) and SMA Nutrition (18.2%) in Dar es Salaam.

### Countries of origin

All products from all four sites were imported and in total, 57.1% (105 out of 184 products) originated from Europe. In Dakar Department and Dar es Salaam, the majority (97.2% and 86.4% of products, respectively) were imported from Europe, while in Phnom Penh and Kathmandu Valley, 43.8% and 14.3% originated from Europe. In Phnom Penh, the country of origin of three products was not given, but of the remaining products, the largest number came from France (21.4%; *n* = 24) with the remainder originating from 14 different countries. In Dakar Department and Dar es Salaam, the products predominantly originated from France (69.4%; *n* = 25) and Ireland (54.5%; *n* = 12), respectively, with the remaining products from both sites originating from three different countries. India was the country of origin of the majority of available CM in Kathmandu Valley (85.7%; *n* = 12), with the rest originating from Ireland.

### Age of introduction

Table 1 shows the wide recommended age/age range provided by manufacturers on CM labels across the four sites. In Phnom Penh the most common recommended age/age range of introduction was 0 to 6 months (14.3%, *n* = 16), closely followed by 0 to 12 months (13.4%, *n* = 15) and 12 to 24 months (11.6%, *n* = 13). In Kathmandu Valley, 0 to 6 and 6 months+ were the most common (28.6%, *n* = 4, respectively). In Dakar Department the recommended age/age range 0 months+, 0 to 6 and 6 to 12 months were each on 22.2% (*n* = 8) of labels. In Dar es Salaam the most common recommended age/age range were 0+ (36.4%, *n* = 8) and 12 to 24 months (22.7%, *n* = 5). Products with an age of introduction between 0 and 6 months were the most common CM in three of the sites, representing over a third of all products (38.4% (*n* = 43) in Phnom Penh; 44.4% (*n* = 16) in Dakar Department; 54.5% (*n* = 12) in Dar es Salaam).

**Table 1:** Recommended age/age range used on labels of commercial milks in Phnom Penh, Kathmandu Valley, Dakar Department and Dar es Salaam

Categorised age of introduction	No. of labels (%)			
	Phnom Penh (*n* = 112)	Kathmandu Valley (*n* = 14)	Dakar Department (*n* = 36)	Dar es Salaam (*n* = 22)
Category* 1 (0–6 months)				
0 (birth–preterm/LBW to 4 kg)	1 (0.9)	0	0	0
0 months+	5 (4.5)	1 (7.1)	8 (22.2)	8 (36.4)
0 to 3 months	2 (1.8)	0	0	0
0 to 6 months	16 (14.3)	4 (28.6)	8 (22.2)	3 (13.6)
0 to 6 months onwards	1 (0.9)	0	0	0
0 to 12 months	15 (13.4)	0	0	1 (4.5)
0 to 5 years	1 (0.9)	0	0	0
0 to 6 years	1 (0.9)	0	0	0
3 to 6 months	1 (0.9)	0	0	0
**Total**	**43** (**38**.**4**)	**5** (**35**.**7**)	**16** (**44**.**4**)	**12** (**54**.**5**)
Category* 2 (6–12 months)				
6 months +	8 (7.1)	4 (28.6)	1 (2.8)	3 (13.7)
6 to 12 months	12 (10.7)	0	8 (22.2)	2 (9.1)
6 to 24 months	6 (5.3)	1 (7.1)	0	0
6 to 36 months	3 (2.6)	0	0	0
9 months+	1 (0.9)	0	0	0
9 to 24 months	1 (0.9)	0	0	0
10 months+	0	0	1 (2.8)	0
10 to 36 months	1 (0.9)	0	5 (13.9)	0
**Total**	**32** (**28**.**6**)	**5** (**35**.**7**)	**15** (**41**.**7**)	**5** (**22**.**7**)
Category* 3 (12–24 months)				
12 months+	10 (8.9)	2 (14.3)	0	0
12 to 36 months	13 (11.6)	0	5 (13.9)	5 (22.7)
1 to 9 years	1 (0.9)	0	0	0
1 to 10 years	1 (0.9)	0	0	0
18 months+	0	2 (14.3)	0	0
**Total**	**25** (**22**.**3**)	**4** (**28**.**6**)	**5** (**13**.**9**)	**5** (**22**.**7**)
Unclear†	l12 (10.7)	0	0	0

* Products were categorised based on the recommended age of introduction as it appeared on the label. When a range was given, the lowest age was used to determine which category the product was placed in, even if the oldest age extended beyond that category. For example, a product labelled 6–24 months was placed in category 2 (6–12 months).

† These were categorised as unclear as all the information on the label was in a language other than English, French or Khmer. Although numbers were written on labels, it could not be assumed what the numbers referred to (months, years or an age category). However, for the purposes of the study, in all other results, these products have been grouped into a category based on their age of introduction being in the 0–6, 6–12 or 12–24 month category, in order to present a complete picture of the results. A combination of Google Translate, numerical values and sometimes an English descriptive name was used to determine which category these products would belong to.

### Age categories

Age categories were used on 84.8%, 92.9%, 63.9% and 86.3% of CM labels in Phnom Penh, Kathmandu Valley, Dakar Department and Dar es Salaam, respectively. The age categories that appeared on more than one label are shown in Table 2. Other terms that appeared on only a single label included ‘2, First steps’; ‘2, for toddlers’; ‘3, 123’; ‘3, Young Explorer’; ‘4, Premature’; ‘Baby’; ‘Toddler’; ‘2, From newborn’ and ‘For toddlers, junior’. Of the products that contained only the number 1 as part of the age category, 100% of labels across all four countries corresponded with the 0–6 month age range. One hundred percent of labels in Phnom Penh, Kathmandu Valley and Dakar Department and 71.4% in Dar es Salaam that contained a number 2 as part of the age category corresponded with 6–12 month age range and the remaining (two) labels corresponded with the 0–6 month age range. Of the products that contained a number 3 as part of the age category, 100% of labels in Kathmandu Valley, Dakar Department and Dar es Salaam, and 95.2% of labels in Phnom Penh corresponded with the 12–24 month age range and the remaining label corresponded with the 6–12 month age range. Seven products (6.3%) in the Phnom Penh sample contained the numbers 1 2 3 in the age category with a corresponding age of introduction of between 12 and 24 months.

**Table 2:** Age categories used on labels of commercial milks in Phnom Penh (*n* = 112), Kathmandu Valley (*n* = 14), Dakar Department (*n* = 36) and Dar es Salaam (*n* = 22)

Age category	No. of labels (%)			
	Phnom Penh	Kathmandu Valley	Dakar Department	Dar es Salaam
1; 1+; Stage 1; Step 1; 1/Newborn	32 (28.6)	4 (28.6)	10 (27.8)	7 (31.9)
2; Stage 2; Step 2	29 (25.9)	5 (35.7)	9 (25.0)	6 (27.4)
3; Stage 3 for toddlers; 3 for older infants	19 (16.9)	2 (14.3)	4 (11.1)	4 (18.2)
No 4 for older infants	0	2 (14.3)	0	0
1 2 3	7 (6.3)	0	0	0
Not applicable	17 (15.2)	1 (7.1)	13 (36.1)	3 (13.6)

### Descriptive names

Table 3 shows the descriptive name categories used to define the associated recommended age category found on CM labels in all four sites. Descriptive names were given on all products in Kathmandu Valley and Dar es Salaam and 87.5% (*n* = 98) and 80.6% (*n* = 29) of products in Phnom Penh and Dakar Department, respectively. The data show that manufacturers make use of a wide variety of descriptive names for a single recommended age category. For example, in Dar es Salaam, manufacturers of CM targeting the 0–6 month recommended age category used five different terminologies to convey a recommended age category (data not shown) and nine different associated descriptive names (Table 3). This trend was noted in all countries except for Nepal, where manufacturers were conservative with their use of descriptive names and corresponding recommended age. Some descriptive names were used across more than one recommended age category; for example, in Phnom Penh, the term FUF was used for products in both the 0–6 month and the 6–12 month recommended age category.

**Table 3:** Descriptive name categories and corresponding recommended age/age range categories appearing on commercial milks* in Phnom Penh, Kathmandu Valley, Dakar Department and Dar es Salaam

Recommended age categories	Descriptive name category*
**Phnom Penh**	
0–6 months†	Infant formula/milk
	Preparation for babies‡
	Milk formula for infants‡
	Premium infant formula
	Powder milk for infants‡
	Soy infant formula
	Super colostrum milk powder
	Super premium
6–12 months†	Follow‐up/on milk*/formula
	Formula milk for further feeding‡
	Premium follow‐on formula
	Follow‐up infant formula
	Soy follow‐on formula
	Infant and toddler formula
12–24 months†	Growing‐up/growth milk‡/formula
	Kid/s formula
	Toddler milk drink
	Older toddler milk drink
	Fortified milk from USA
	Milk from USA
	Soy growing‐up formula
	Nutritious milk drink
	Nutritional supplement
	Premium milk drink
**Kathmandu Valley**	
0–6 months†	Infant formula/milk
	Infant milk substitute
	Extra hungry infant milk
6–12 months†	Follow‐up/on formula/milk
	Follow‐up/on formula/milk, complementary food
12–24 months†	Follow‐up/on formula/milk
**Dakar Department**	
0–6 months†	Infant formula/milk
	Milk for infants‡
	Starter infant formula
	Premium starter infant formula
6–12 months†	Follow‐up/on milk/formula
	Follow‐on milk/formula for infants‡
	Premium follow‐up formula
12–24 months†	Growing‐up/growth milk‡/formula
	Growing‐up milk formula
**Dar es Salaam**	
0–6 months†	Starter infant formula
	Infant starter formula
	Infant milk for hungrier babies
	Hungry milk
	Infant formula/milk
	Extra hungry infant milk for hungrier babies
	First milk
	First infant milk; Breastmilk Substitute
	Soy (Protein) infant formula
6–12 months†	Follow‐up/on milk/formula
12–24 months†	Growing‐up milk/formula
	Toddler milk
	Soy formula
	Follow‐on formula for young children, Growing‐up milk

* Commercial milks for special dietary or medical purposes not included.

† Products were categorised based on the recommended age of introduction as it appeared on the label. When a range was given, the lowest age was used to determine which category the product was placed in, even if the oldest age extended beyond that category. For example, a product labelled 6–24 months was placed in category 2 (6–12 months).

‡ Descriptive name in English was provided by a professional translation of the original label text.

### Use of the word formula

Table 4 represents how labels from each age category, across all four sites, described the product using the word ‘formula’.

**Table 4:** Number and percentage of products in each age category that described the product using the word ‘formula’ on the label in Phnom Penh, Kathmandu Valley, Dakar Department and Dar es Salaam

Site	Total sample	Age category		
		0–6 months*	6–12 months*	12–24 months*
	No. of labels (%†)	No. of labels (%†)	No. of labels (%†)	No. of labels (%†)
Phnom Penh	75 (67.0)	34 (72.3)	26 (76.5)	15 (48.4)
Kathmandu Valley	12 (85.7)	4 (80.0)	4 (80.0)	4 (100.0)
Dakar Department	17 (47.2)	9 (56.3)	7 (46.7)	1 (20.0)
Dar es Salaam	13 (59.1)	6 (50.0)	4 (80.0)	3 (60.0)

* Products were categorised into groups based on the recommended age/age range of introduction that appeared on the label. When an age range was given, the lowest age was used to determine which category the product was placed in, even if the upper end of the age range extended beyond that category. For example, a product labelled 6–24 months was placed in category 2 (6–12 months).

† Percentages are according to the number of labels in each category.

### Image of a bottle on the label

**Figure 2:**
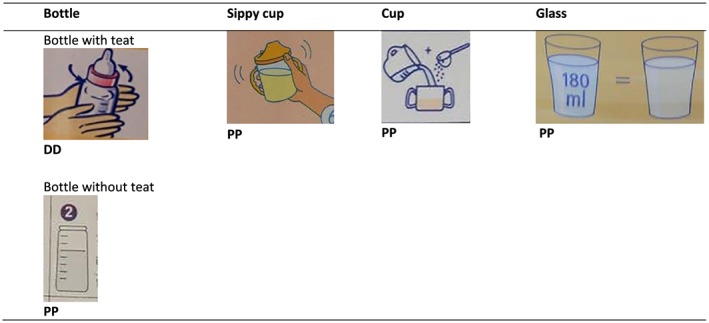
Examples of images of a feeding bottle, sippy cup, cup, glass depicted on the labels of CM from Phnom Penh, Kathmandu Valley, Dakar Department and Dar es Salaam.

Labels were assessed for the presence of an image of a feeding bottle (with or without a teat) and/or a sippy cup 3Defined in the Merriam Webster dictionary as a spill‐proof cup that usually has a detachable lid with a spout and is designed for young children (Merriam Webster dictionary 2015)./cup/glass. See Fig. 2 for image examples. The use on the label of an image of a feeding bottle decreased with an increase in age category, and the use of an image of a sippy cup/cup/glass increased with an increase in age category across all study sites, with the exception of the 12–24 month age range in Kathmandu Valley and Dar es Salaam (Table 5). An image of a feeding bottle was found on all or some labels across all the age categories in each study site.

**Table 5:** Labels of commercial milks from Phnom Penh, Kathmandu Valley, Dakar Department and Dar es Salaam depicting a feeding bottle, sippy cup, cup and glass

Age category	Images used	No. of labels (%†)			
		Phnom Penh	Kathmandu Valley	Dakar Department	Dar es Salaam
0–6 months*	*n*	*47*	*5*	*16*	*12*
	Bottle/teat	44 (93.6)	5 (100)	12 (75.0)	9 (75.0)
	Sippy cup/cup/glass	2 (4.3)	0	4 (25.0)	1 (8.3)
	None	1 (2.1)	0	0	2 (16.7)
6–12 months*	*n*	*34*	*5*	*15*	*5*
	Bottle/teat	27 (79.4)	4 (80.0)	10 (66.7)	1 (20.0)
	Sippy cup/cup/glass	7 (20.6)	1 (20.0)	4 (26.7)	3 (60.0)
	None	0	0	3 (20.0)	2 (40.0)
12–24 months*	*n*	*31*	*4*	*5*	*5*
	Bottle/teat	11 (35.5)	4 (100)	1 (20.0)	1 (20.0)
	Sippy cup/cup/glass	13 (41.9)	0	3 (60.0)	3 (60.0)
	None	7 (22.6)	0	1 (20.0)	1 (20.0)

* Products were categorised into groups based on the recommended age of introduction that appeared on the label. When an age range was given, the lowest age was used to determine which category the product was placed in, even if the upper end of the age range extended beyond that category. For example, a product labelled 6–24 months was placed in category 2 (6–12 months).

† Percentages are according to the number of labels in each category.

### Cross‐promotion 4Cross‐promotion also commonly known as ‘brand cross‐over promotion’, ‘brand‐stretching’ or ‘line extension’, broadly refers to the practice of one product being used to advertise another (Park *et al*. 1991; Berry *et al*. 2010).


In the four sites the results show that the majority of manufacturers of CM, which manufacture IF, for the 6–24 month age range, displayed at least one example of cross‐promotion of a FUF/GUM with one or more of the company's IF (Table 6). In Phnom Penh 61 of the 63 products (96.8%) that were recommended for the 6–24 month age range were manufactured by a company that also produced an IF, and 96.7% of these cross‐promoted. In Kathmandu Valley, all products with a 6–24 month age of introduction (*n* = 9) were manufactured by a company that also had an IF available, and all cross‐promoted. In Dakar Department and Dar es Salaam, 93.3% and 90.0% of products for the 6–24 month age category, respectively, cross‐promoted with an IF made by the same manufacturer. The two products in Phnom Penh and one product each in Dakar Department and Dar es Salaam that did not display cross‐promotion with an IF, manufactured by the same company, were products with a 12–24 month age of introduction.

**Table 6:** Cross‐promotion of products for the 6–24 month age range with an infant formula produced by the same manufacturer in Phnom Penh, Kathmandu Valley, Dakar Department and Dar es Salaam

Product category	Cross‐promotion categories	No. of labels (%†)			
		Phnom Penh	Kathmandu Valley	Dakar Department	Dar es Salaam
6–12 months*	Similar or same colour schemes/designs used as manufacturer's IF	32 (94.1)	5 (100)	10 (66.7)	5 (100)
	Similar or same brand name as manufacturer's IF	32 (94.1)	5 (100)	10 (66.7)	5 (100)
	Similar or same slogans/mascots/symbols used as manufacturer's IF	29 (85.3)	1 (20.0)	10 (66.7)	2 (40.0)
12–24 months*	Similar or same colour schemes/designs used as manufacturer's IF	26 (83.9)	4 (100)	4 (80.0)	4 (80.0)
	Similar or same name used as manufacturer's IF	27 (87.1)	4 (100)	3 (60.0)	4 (80.0)
	Similar or same slogans/mascots/symbols used as manufacturer's IF	24 (77.4)	0	3 (60.0)	3 (60.0)

* Products were categorised into groups based on the recommended age of introduction that appeared on the label. When an age range was given, the lowest age was used to determine which category the product was placed in, even if the upper end of the age range extended beyond that category.

† Percentages given are those excluding manufacturers that do not sell other commercial milks for 6–12 months.

**Figure 3:**
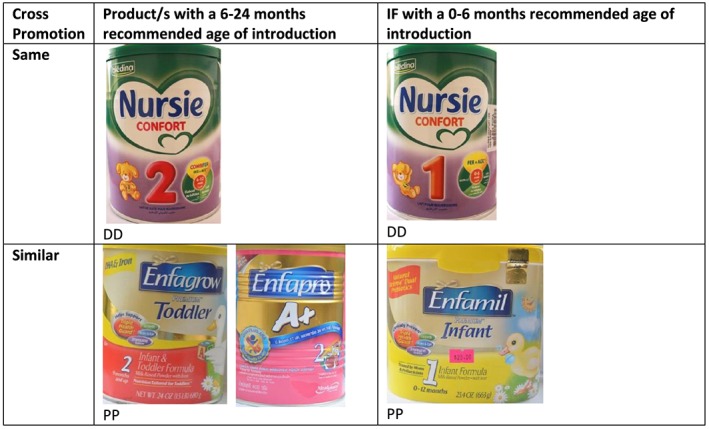
Front‐of‐pack shots displaying cross‐promotion using the same or similar label elements between a product with a recommended age of introduction of 6‐24 months and an IF.

Examples of products that cross‐promoted are provided in Fig. 3 and illustrate how two or more products can cross‐promote by sharing many label elements – colour scheme/design/layout; font types and colours; brand/sub‐brand; slogan/mascot/symbols.

The use of pack‐shots 5A pack‐shot refers to an image of a product produced by the manufacturer. and/or text reference to an IF on the label of a product with an age of introduction from 6 to 24 months was a less prevalent cross‐promotion practice. In Phnom Penh, 15.6% of products with an age of introduction between 6 and 24 months included a pack‐shot of an IF on the label, while this practice was not found in Kathmandu Valley, Dakar Department or Dar es Salaam. In Phnom Penh, 58.8% of the labels of products with an age of introduction of 6–24 months included a textual reference to an IF that was usually in the form of referring to the manufacturer's total range of products that included IF. In Kathmandu Valley, Dakar Department and Dar es Salaam, 20%, 13.3% and 40% of products with a recommended age of introduction of 6–24 months, respectively, provided a textual reference to an IF made by the same manufacturer.

## Discussion

This study found that many of the characteristics – including age of introduction, descriptive names, use of the term formula and images – on the labels of FUF and GUM are the same or similar to IF, which justifies categorising them as breastmilk substitutes as they are clearly promoted as such. In addition, the study highlights that cross‐promotion between FUF, GUM and IF manufactured by the same company is common. These practices need to be prohibited in the interest of protecting and promoting optimal breastfeeding and IYCF practices. The CM industry is becoming increasingly globalised, and currently the global top five CM manufacturers – Nestlé, Danone, Mead Johnson, Abbott and Heinz – account for 56% of the world CM market (Coriolis 2014). Many reports show that CM is a growing market in LMIC (Bandy 2014; Coriolis 2014; UBIC Consulting 2014). All the CM products found in the four study sites (two in Africa and two in Asia) were imported. Nestlé products were found in all four sites, Danone in three (Kathmandu Valley, Dakar Department and Dar es Salaam), Abbott in two (Phnom Penh and Dar es Salaam) and Mead Johnson and Heinz in one (Phnom Penh). However, products from many additional CM manufacturers (25 in Phnom Penh) were also found, illustrating that attention and reporting should not only be on the practices of the well‐known manufacturers.

Findings from this study highlight that manufacturers of FUF/GUM provide a diverse and confusing range of recommended age of introduction on product labels. This is considered inappropriate considering that the WHA, WHO and Codex have unequivocally stated that FUF/GUM are unnecessary (WHO 1986; WHO 2013; Codex Alimentarius 2014), and not misleading the consumer is a basic principle of the Global Strategy for IYCF, Codex and most national regulations.

The CM available in the four countries in this study also provided a wide range of age categories, either in words or using a numbered stage, in addition to giving a recommended age/age range of introduction. These categories are not standardised and potentially lead to confusion. For example, numbered stages that use only numerals (e.g. 1, 2 or 3) may be interpreted to reflect an age (in months or years) rather than a stage. As a consequence, a mother may select an inappropriately formulated product for the age of her infant/young child (e.g. an FUF with an inadequate nutrient composition for a child of less than 6 months). An Italian study showed that when products had a similar layout but depicted different numbers, highly educated pregnant women and mothers were confused by the use of numerals on labels (e.g. Aptamil 2). Only 43% of mothers were able to assign the correct meaning, in terms of age of use, after careful reading of an FUF advertisement, and the remainder assigned numerous meanings to the numeral including ‘for the second phase of growth’, ‘2 cups’, ‘better than 1’, ‘added value’, ’2‐month‐old’ or ‘2‐year‐old babies’ (Cattaneo *et al*. 2014). In this study, numbered stages were generally found to represent specific age recommendations, for example, ‘stage 2’ featured predominantly on products with a recommended 6–12 month age of introduction. However, in Phnom Penh, 6.3% of products used the age category ‘1 2 3’, which could be confusing to consumers and further illustrates the potential for confusion.

The use of numbered stages suggests that CM manufacturers intend all products from 0 to 24 months to be seen as a group with sub‐divisions. This could contribute to the perception that products from 6 to 24 months are ‘formula’ and part of the same group of CM products that include those for infants 0–6 months of age (IF), and this would imply that they are suitable for use as a substitute for breastmilk (Berry *et al*. 2010).

The wide range of descriptive names on the labels of CM products and the origin of those names is also problematic because this can lead to confusion among mothers and caregivers. An Australian study showed that women perceive GUM to form part of a line of products (that includes IF and FUF), which they referred to collectively as ‘formula’ (Berry *et al*. 2010). These women believed that the appropriate use of ‘formula’ products would be to replace breastfeeding. For example the terms ‘growing‐up milk’ (GUM) and/or ‘toddler milk’ (which appear to originate from manufacturers) emerge as a subcategory of what Codex includes within the scope of the FUF standard (Codex Alimentarius 1987). Products termed GUM typically have a recommended age of introduction from 12 months, while the Codex term FUF usually appears in conjunction with a 6–12 month recommended age of introduction. Various other inconsistencies between label elements, such as descriptive name with recommended age of introduction, and conflicting descriptive name on different sections (e.g. front and back panel) of the label were common. The findings underscore the importance of the current Codex review of the FUF standard not only in addressing differences in the composition of FUF with a 6–12 and 12–36 months differentiation but also to consider descriptions and labelling matters, in line with the fact that Codex itself, together with the WHO, has stated that these products are unnecessary (Codex Alimentarius 2014).

In this study the word ‘formula’ was commonly used on labels to describe all CM from 0 to 24 months in all four countries. The use of the word ‘formula’ is a practice that could contribute to the perception that the product is a substitute for breastmilk and confirms that all CM are intended to be used as breastmilk substitutes and so should be considered as such and be subject to Code compliance. This is further supported by the finding that feeding bottles were displayed not only on the labels of CM for infants 0–6 months of age (IF) but also on CM from 6 to 24 months (FUF/GUM). The use of images of feeding bottles on products with an age of introduction of between 6 and 24 months contributes to the perception that FUF/GUM and IF are suitable for use as a substitute to breastmilk (Berry *et al*. 2010). This practice requires these products to comply with the Code and that they are therefore in contravention of the Code. As Codex is currently reviewing the FUF standard, they should fulfil their mandate, in WHA resolution 54.2, to take the Code and relevant subsequent World Health Assembly resolutions into consideration when developing standards and guidelines and should therefore provide specific text to ensure all CM are referred to as breastmilk substitutes and subject to the Code (WHO 2001).

FUF/GUM packaging is commonly used to promote IF, and cross‐promotion between FUF/GUM and IF was found on almost all CM products for the ages 6–24 months manufactured by companies that also produce an IF. This is suggestive that manufacturers use cross‐promotion as a deliberate strategy to evade restrictions on the marketing of IF and that it is their intention for IF, FUF and GUM products that they manufacture to be perceived as a single group. Research has shown that mothers do not differentiate between CM categories (Faircloth *et al*. 2007; Berry *et al*. 2010) and that cross‐promotion results in consumers associating characteristics advertised by one product with all similar products produced by the same manufacturer (Berry *et al*. 2010; Berry *et al*. 2012a, Berry *et al*. 2012b). The United Kingdom provides an example of country legislation that specifically prohibits the cross‐promotion of IF and FUF by stating that ‘Infant formula and follow‐on formula shall be labelled in such a way that it enables consumers to make a clear distinction between such products so as to avoid any risk of confusion between infant formula and follow‐on formula’ (Department of Health England 2007)

## Conclusion and recommendations

Global guidance from normative bodies forms the basis of most recommendations and regulations in LMIC and should provide detailed and specific guidance. The WHO has, at the request of the 65th World Health Assembly (WHA 2012), drafted guidance to provide both clarification and guidance on the inappropriate promotion of foods for IYC in the form of a ‘Discussion Paper: Clarification and Guidance on Inappropriate Promotion of Foods for Infants and Young Children’ that was published for comment in July 2015 for final presentation at the WHA in May 2016. Its recommendations include that the implementation of the Code should ‘clearly cover all products that function as breastmilk substitutes’ and the prohibition of many practices observed in this study (WHO 2015). Such guidance is, based on this research, necessary and should be supported towards adoption of a WHA resolution, in order to protect and promote breastfeeding and optimal IYCF practices.

## Source of funding

Funding was received from the Bill & Melinda Gates Foundation (BMGF). The authors affirm their independence from the funder. The funder played no part in the study design, collection, analysis or interpretation of data, or in the writing of the report, or in the decision to submit the article for publication.

## Conflicts of interest

Jane Badham and Rosalyn Ford have acted as nutrition consultants to the food industry but have not worked for manufacturers of breastmilk substitutes or complementary foods.

## Contributions

LS, JB and EZ conceptualised and CP and RF designed the study. CP and RF entered, and CP, RF and AF analysed and interpreted the data with assistance from LS and JB. CP, RF, AF, LS and JB drafted the paper. EZ critically reviewed the paper.
